# When Load Does not Tell the Whole Story: Acute Effects of Effort Matched Resistance Training Protocols on Arterial Stiffness

**DOI:** 10.1002/ejsc.70164

**Published:** 2026-03-24

**Authors:** Eleftherios Karanasios, James Faulkner, Scott Hannah

**Affiliations:** ^1^ Faculty of Health & Wellbeing School of Sport Health and Community University of Winchester Winchester UK; ^2^ Primary Care Research Centre University of Southampton Southampton UK

**Keywords:** central haemodynamics, proximity to failure, pulse wave velocity, repetitions in reserve

## Abstract

This study examined acute vascular responses to three resistance training (RT) protocols differing in load and volume but matched for proximity to failure. Eleven adults (6 males and 5 females) performed three RT protocols consisting of the hexagonal deadlift and bench pull exercises with the following: (i). Low‐volume, moderate‐load, and high‐repetition (Condition A: 2 × 10 repetitions), (ii). Low‐volume, high‐load, and low‐repetition (Condition B: 2 × 4 repetitions), and (iii). Moderate‐volume, high‐load, and low‐repetition (Condition C: 5 × 4 repetitions). Proximity to failure was set at 2 repetitions in reserve. Measurements of carotid‐femoral pulse wave velocity (cfPWV) and pulse wave analysis (e.g., augmentation index) were collected at baseline, immediately post, and 15 min posttraining, whereas muscle oxygenation was monitored during performance the hexagonal deadlift. Condition A induced significantly greater increases in cfPWV (6.2 ± 0.6 to 6.9 ± 0.8 m/s) when compared to both Condition B (6.5 ± 0.8 to 6.4 ± 0.7 m/s) and Condition C (6.3 ± 0.8 to 6.4 ± 0.6 m/s), (all *p* < 0.05). No significant changes were observed in muscle oxygenation variables across conditions. These findings suggest that the number of repetitions per set and total time under tension (Condition A), rather than absolute load or set volume, play a more important role in mediating acute hemodynamic responses following RT.

## Highlights

- A moderate‐load high‐repetition resistance training protocol acutely increased arterial stiffness markers more than heavy‐load low‐repetition protocols, despite matched proximity to failure set and repetition volume.
- The number of repetitions per set and the associated time under tension seems to be better determinants of acute vascular responses than total training volume or absolute load.
- Muscle oxygenation did not significantly differ between protocols, suggesting that acute macrovascular changes may occur independent of localized tissue perfusion dynamics.

## 1 Introduction

Arterial stiffness (AS) is an independent determinant of cardiovascular disease (CVD), closely linked to hypertension and future cardiovascular events (Kim and Kim 2019). Carotid‐femoral PWV (cfPWV) (the speed at which the pressure wave propagates from the carotid to the femoral artery) is the gold standard measurement of AS (Kim and Kim 2019). Resistance training (RT) is currently recognized as an effective intervention for the prevention and management of CVD, offering a wide range of health benefits (Paluch et al. 2024). Yet, its relationship with AS remains ambiguous with conflicting findings reported (Karanasios et al. 2023). Differences in the loading characteristics, including variations in volume, loading intensity, and proximity to failure, could explain the inconsistencies observed.

Volume is a key variable in RT prescription determining training‐induced physiological adaptations (Correia et al. 2023; Figueiredo et al. 2015). Volume quantification methods, such as volume load (set x reps x load), repetition (number of reps), and set volume (number of sets), are all considered viable options, since there is no consensus on a single metric (B. Schoenfeld and Grgic 2018). Guidelines suggest 2–12 weekly sets per muscle group (Ratamess et al. 2009); however, recent evidence indicates that < 5 weekly sets are sufficient to improve strength and hypertrophy, enabling time‐efficient training alternatives (Androulakis‐Korakakis et al. 2020; B. J. Schoenfeld et al. 2017). Most studies examining the acute effects of RT on AS have adopted protocols consisting of ≥ 3 sets per exercise (Karanasios et al. 2023). Given that acute training variables, such as volume, dictate physiological responses to RT and that time constraints are a main barrier to RT (Hoare et al. 2017), it seems crucial to explore the influence of RT volume on AS.

Previous research has mostly used a fixed number of per‐set repetitions (e.g., 10 repetitions at 70% of 1RM) (Kingsley et al. 2017; Parks et al. 2020). This method, although dominant in RT prescriptions, fails to adequately address proximity to failure, individual variability in the number of repetitions performed at given relative loads and daily fluctuations in human performance (Bartholomew et al. 2008; Cooke et al. 2019). Recent evidence indicates that proximity to failure can significantly affect AS independent of load, volume or rest interval length (Karanasios, Hannah, et al. 2025b). Along this line, the repetitions in reserve (RIR) based rating of perceived exertion (RPE) scale was developed to quantify and equalize effort per set across trainees and autoregulate load selection according to daily readiness (Helms et al. 2016; Zourdos et al. 2016).

We previously showed that when effort is equated (i.e., both protocols performed to volitional failure) moderate loads with higher repetitions promote a greater increase in cfPWV compared to higher loads‐lower repetitions RT schemes (Karanasios, Hannah, et al. 2025a). However, in the abovementioned study, volume was not equated between the experimental protocols and each set was performed to volitional failure. It is now acknowledged that performing RT to failure is not required to induce strength or hypertrophic adaptations (Grgic et al. 2022; Vieira et al. 2021). Thus, considering the above limitations of the available literature, it is important to assess how different loading intensities at submaximal effort mediate AS.

Moreover, the exact mechanisms explaining AS changes following acute RT are not entirely clear (Figueroa et al. 2019). Evaluation of microvascular function can provide a better understanding of macrovascular responses during RT. NIRS derived measurements of muscle oxygenation such as parameters of tissue saturation index (TSI%) have been positively correlated with macrovascular function (*r* = 0.62) following reactivity protocols indicating an interconnection between macro and microcirculation (Laurent et al. 2022; Pinheiro et al. 2023). Different loading parameters may alter intramuscular oxygen dynamics and modulate microvascular perfusion, potentially contributing to macrovascular responses that can influence AS.

The purpose of this study was twofold: (i). To compare the effects of different loads on AS and muscle oxygenation between two protocols performed at submaximal effort while having set and repetition volume, proximity to failure, and rest interval matched and (ii). To compare the effects of set volume on AS and microvascular reactivity between a low versus moderate volume while having load, proximity to failure, and rest interval duration equated.

It was hypothesized that: (i). The heavy load‐low repetition RT protocols will promote a more pronounced increase in AS indices than the moderate load‐high repetition RT protocol. (ii). The RT protocol with higher set volume will promote a more pronounced increase in measures of AS than the ones with lower set volume.

## 2 Methods

### 2.1 Participants

Eleven young healthy participants (6 male and 5 female) participated in this study. Participants had been participating in RT at least once a week for the last 6 months. All participants were healthy, nonhypertensive, and free of any cardiovascular, musculoskeletal, and metabolic disease. Female participants indicated testing during the follicular phase of their menstrual cycle, although prior studies suggest minimal menstrual cycle effects on AS as measured by cfPWV (Priest et al. 2018). All participants provided written informed consent prior to data collection. The study received approval from the Faculty of Health and Wellbeing at the University of Winchester (HWB_REC_240_Karanasios).

Sample size was calculated using G*Power 3.1 (version 3.1.9.7, Heine University, Düsseldorf, Germany). For a repeated measures within factors design with an effect size *F* = 0.45, based on (Karanasios, Hannah, et al. 2025a), a significance level of 0.05, a power of 0.80, and a correlation among repeated measurements of 0.5., a sample size of 11 participants was deemed appropriate to detect differences between the experimental conditions while accommodating a 10% dropout rate.

**FIGURE 1:**
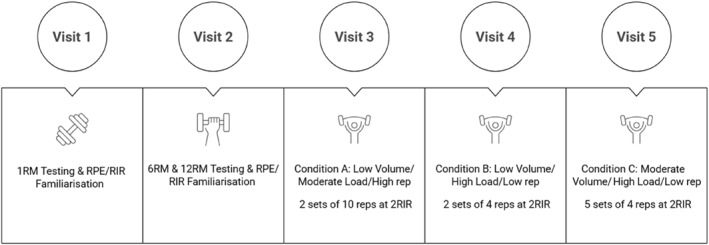
Schematic representation of the testing procedure. Visits 3, 4, and 5 performed in a randomized order.

Participants took part in a randomized cross over design and reported to the research facility on five occasions (Figure 1). The initial two visits involved anthropometric assessments, familiarization with experimental procedures, and repetition maximum testing to determine individual 1, 6, and 12RM loads for the hexagonal deadlift (HDL) and bench pull (BP) exercise. Participants were asked to refrain from strenuous physical activity 24 h prior to testing and from caffeine consumption 4 h before each testing session (Townsend et al. 2015).

Visits three, four, and five were the experimental trials which incorporated two exercises: HDL and BP, with the HDL being selected due to its superior engagement of the vastus lateralis relative to the conventional deadlift, and both exercises necessitating minimal spotting (Camara et al. 2016; Ronai and Taber 2021). The BP exercise was performed using a slightly inclined bench to allow participants to pull the barbell with elbows fully extended. The order of exercises was not randomized. All participants performed the hexagonal deadlift first, followed by the bench pull. A minimum of 24 h was provided between experimental sessions.

### 2.2 Familiarization Sessions (Visits 1 and 2)

During the first visit, 1RM testing for the HDL and BP was conducted following the guidelines of National Strength and Conditioning Association (Haff and Triplett 2015). Visit two involved 6RM and 12RM assessments for the same exercises. The maximum amount of weight lifted for 1, 6, and 12 repetitions with proper form in the HDL and BP exercise was recorded as the participant's 1, 6, and 12RM load, respectively. Opening weights for RM attempts were based on self‐reported training history. All RM loads were determined in no more than five attempts with rest periods of 4 min between the tests.

During the familiarization sessions, participants were provided with detailed instructions on the proper utilization of the RPE/RIR scale. They were informed that an RIR of 0 signifies maximal effort, corresponding to an RPE of 10, whereby no further repetitions could be completed without external assistance. Similarly, an RIR of 2 indicates the ability to perform two additional repetitions corresponding to an RPE of 8. Participants familiarized using the RPE/RIR scale during all the warm‐up sets throughout the familiarization and experimental sessions. To enhance RIR's predictive accuracy, participants were also educated on the distinction between perceived effort and discomfort. Specifically, they were informed that repeated maximal contractions to failure (e.g., 12RM) produce more discomfort than a single maximal contraction (e.g.., 1RM) even though the effort level remains the same (i.e., maximal) (Smirmaul 2012). They were briefed to conceptualize effort as the ‘physical and mental energy invested in the exercise performed’, and discomfort as the ‘physiological and unpleasant sensations experienced during performance of the exercise’ (Fisher et al. 2017; Marcora 2009). Both visits 1 and 2 served as a familiarization procedure for RIR estimation and as reference points for load selection in the subsequent visits (Refalo et al. 2024).

### 2.3 Experimental Sessions (Visits 3, 4, and 5)

In the subsequent three visits, participants were allocated to one of the three experimental protocols in a randomized order (https://www.randomizer.org/). Participants were instructed to self‐select a load that they perceived they could complete the predetermined number of repetitions (4 or 10), while maintaining 2 RIR upon completion of the 4th and 10th repetition, respectively. Two warm‐up sets were completed prior to testing, and participants reported their perceived RIR based on the selected load. Only during the first set was the load selected by participants; in subsequent sets, the load was adjusted from the researcher based on the RPE/RIR reported during the first warm‐up set and through consultation of data collected from visits 1 and 2 (Helms et al. 2017). The testing protocols consisted of the following:

Low Volume/Moderate Load/High Repetition (Condition A): 2 sets x 10 reps at 8RPE/2RIR.

Low Volume/High Load/Low Repetition (Condition B): 2 sets x 4 reps at 8RPE/2RIR.

Moderate Volume/High Load/Low Repetition (Condition C): 5 sets x 4 reps at 8RPE/2RIR.

Volume classification was based on the clustering proposed by B. J. Schoenfeld et al. (2017) and considered as follows: low < 5 weekly sets, moderate 5–9, and high ≥ 10 weekly sets, since no clear consensus exists about this metric (B. J. Schoenfeld et al. 2017).

Rest duration was 2 minutes between sets and between exercises for all conditions. Participants were guided by the researcher to adopt a repetition tempo of approximately 1s concentric, 2s eccentric, and 1s isometric pause between concentric and eccentric actions.

Hemodynamic measurements were obtained three times: before training (baseline), immediately post (Post), and 15 min post training (15Post) in a supine position. The initial data collection occurred after a 10‐min supine rest. Upon completion of the acute RT protocol, participants returned to the supine position while the data collection apparatus was applied. Post and 15Post hemodynamic measurements were obtained at 1 min and 15 min following the acute exercise intervention.

### 2.4 Hemodynamic Measurements

All hemodynamic measurements were performed using an oscillometric cuff‐based device (Vicorder, Skidmore medical, Bristol, UK), that computes pulse wave velocity by simultaneously recording the upstroke of the femoral and carotid pulsations. Validity and reliability of the Vicorder device has been elsewhere established (Hickson et al. 2009; Teren et al. 2016). Two inflatable cuffs were used to measure carotid‐femoral pulse wave velocity (cfPWV), one cuff positioned around the neck over the carotid artery and the other encircling the thigh over the femoral artery. CfPWV was calculated by dividing the pulse wave travel distance by the pulse transit time between the two recording sites. Transit time was determined by the software using an in‐built cross‐correlation algorithm and travel distance length was defined as the distance from the suprasternal notch to the mid upper thigh cuff, as indicated by the manufacturer. cfPWV measurements were conducted in duplicate and averaged. If the cfPWV values differed by more than 0.5 m/s, a third measurement was performed, and the median cfPWV value was recorded (Van Bortel et al. 2012). AIx is an indicator of AS which reflects the augmentation of systolic blood pressure by reflection of the peripheral pulse wave. AIx was calculated as the ratio between the augmentation pressure (AP) and the central pulse pressure (cPP) and was expressed as a percentage (AIx = AP/cPP x 100). AIx was adjusted to a heart rate of 75 bpm (AIx@75) to decrease its reliance on HR, as previously recommended (Wilkinson et al. 2000). Measurements of subendocardial viability ratio (SEVR), an index representing myocardial perfusion, which is calculated as the ratio between diastolic pressure time index (DPTI) and systolic pressure time index (SPTI) (Tsiachris et al. 2012), and of central and peripheral blood pressures were conducted utilizing a cuff positioned around the upper arm over the brachial artery and evaluated through device‐specific pulse wave analysis (Baier et al. 2018).

### 2.5 Oxygenation Measurements

Tissue oxygenation of the vastus lateralis was monitored continuously during the experimental protocols using a portable NIRS device (Portamon, Artinis Medical System, BV, The Netherlands). The validity and reliability of this device during RT has been previously established (Scott et al. 2014). NIRS devices operate using a probe emitting near‐infrared light into muscle tissue. This light penetrates the skin and subcutaneous layers, reaching the underlying muscle, where it is differentially absorbed by oxygenated (O_2_Hb) and deoxygenated hemoglobin (HHb). NIRS software receives this information via Bluetooth connection and quantifies the relative changes in O_2_Hb and HHb. Total hemoglobin (tHb = *O*_2_Hb + HHb) was derived as an index of local blood volume, whereas the tissue saturation index (TSI%)—calculated automatically as O_2_Hb/tHb x 100—reflected the balance between oxygen delivery and consumption in the microvasculature (Alvares et al. 2020). These variables represent the combined absorption changes of hemoglobin and myoglobin within the illuminated tissue volume (Barstow 2019). The NIRS probe was attached on the longitudinal axis over the lower third of the vastus lateralis muscle of the participant's dominant thigh as described for EMG electrodes placement (Ebben et al. 2009). The probe was covered in transparent plastic to prevent direct contact with the skin and to mitigate probe movement. An elastic bandage was then wrapped around the probe to prevent pollution of ambient light and further minimize unwanted movement. Using the Oxysoft software (Oxysoft, Artinis Medical Systems, BV, The Netherlands), data were sampled at 50 Hz. The variables considered for analysis from the tHb and TSI% parameters were the following: Baseline TSI% (TSI%base) and Post TSI% (TSI%post) calculated as the average of 30s prior to and post exercise respectively; tHb and TSI% reperfusion rate calculated as the upslope of the 10s window immediately following the end of the exercise.

### 2.6 Statistical Analysis

All descriptive data are expressed as means ± standard deviation. The normality of the distribution was evaluated utilizing the Shapiro–Wilk test in conjunction with visual inspection. A two‐way (Condition vs. Time) repeated measured analysis of variance (ANOVA) was conducted to evaluate the effects of condition, time, and the interaction between them for: cfPWV; AIx; AIx@75; central systolic blood pressure (cSBP); central diastolic blood pressure (cDBP); peripheral systolic (pSBP); peripheral diastolic (pDBP); augmentation pressure (AP); central mean arterial pressure (cMAP); central pulse pressure (cPP), SEVR and TSI%. A one‐way ANOVA was also performed to detect differences in the tHb and TSI% upslopes between the experimental conditions. Violations of sphericity were adjusted using Greenhouse–Geisser. Post hoc pairwise comparisons with a Bonferroni correction were conducted when Condition by iime interactions were detected. The effect size analysis was evaluated using the partial Eta squared ($\eta_{p}^{2}$). The magnitude of effect size was interpreted as trivial (< 0.01), small (0.01–0.06), moderate (> 0.06–0.14), and large (> 0.14) (Richardson 2011). Significance was set at *p* < 0.05. Data were analyzed using SPSS version 28 statistical software (SPSS Inc., Chicago, IL, USA). A linear mixed‐effects model (jamovi, version 2.3.28) was additionally employed to evaluate the impact of the intervention on cfPWV (the primary outcome), while controlling for cMAP as a covariate (Townsend et al. 2015). Random intercepts were incorporated for each individual to accommodate the repeated measurements across time. Fixed effects within the model included condition, time, and the interaction between condition and time. cMAP was included as a covariate to mitigate its potential influence on cfPWV. Residual plots were scrutinized to validate the model assumptions relating to normality and homoscedasticity.

## 3 Results

Participant's demographics and protocol's descriptives are displayed in Table 1.

**TABLE 1:** Participant demographics and RT protocol's descriptives.

		Condition A	Condition B	Condition C
Age (y)	32.6 ± 11.6			
Weight (kg)	72.8 ± 17.8			
Height (cm)	173.6 ± 5.5			
1RM HDL	91.1 ± 35.3			
1RM BP	54.6 ± 16.1			
% 1RM HDL		63.4	84.7	83.9
% 1RM BP		68.2	85.0	84.0
Volume (repetitions)		20	8	20
Volume load HDL (kg)		1154.5 ± 471.9	616.7 ± 245.1	1527.3 ± 625.2
Volume load BP (kg)		745.5 ± 208.4	371.6 ± 111.9	918.2 ± 280.2
RPE HDL		8.0 ± 0.3	8.0 ± 0.4	8.0 ± 0.3
RPE BP		7.9 ± 0.5	7.9 ± 0.4	8.0 ± 0.4

*Note:* Volume load is calculated as follows: sets x repetitions x load. Data presented are mean ± SD.

Abbreviations: BP: bench pull; HDL: hexagonal deadlift; RPE: Rate of perceived exertion; RM: repetition maximum.

Hemodynamic and oxygenation data are shown in Tables 2 and 3, respectively. Significant condition and time interactions were detected for cfPWV $(\eta_{p}^{2}=0.59),$ AIx $(\eta_{p}^{2}=0.42)$ cPP$(\eta_{p}^{2}=0.23)$, AP $\left(\eta_{p}^{2}=0.49\right)$, cDBP, $\left(\eta_{p}^{2}=0.22\right)$, and pDBP $(\eta_{p}^{2}=0.22),$ (all *p* < 0.05). Post hoc analyses revealed that increases in cfPWV, AIx, cPP, and AP from BL at post were significantly higher in Condition A than both Condition B and Condition C. In addition, reductions in cDBP and pDBP from BL values to post were significantly greater in Condition A than Condition B and Condition C. cMAP had no significant main effect on cfPWV (*p* = 0.83), therefore a similar statistical condition by time interaction was observed for cfPWV when controlling for cMAP (*p* = 0.007).

**TABLE 2:** Hemodynamic variables.

	Time point			Interaction effect	
	Baseline	Post	15Post	*p* for interaction effect	Effect size (η^2^_p_)
cSBP (mmHg)^c^					
Condition A	113.4 ± 11.5	123.5 ± 11.8	114.4 ± 13.4	0.72	0.04
Condition B	113.7 ± 11.5	120.3 ± 15.2	113.7 ± 13.5		
Condition C	112.5 ± 12.7	120.5 ± 12.5	114.8 ± 13.0		
cDBP (mmHg)^a^					
Condition A	58.3 ± 9.3	47.9 ± 13.5	53.9 ± 10.4	0.03	0.22
Condition B	62.4 ± 9.2	58.7 ± 13.1	57.5 ± 9.7		
Condition C	62.3 ± 10.8	60.0 ± 9.9	61.7 ± 8.6		
cfPWV (m/s)^a^					
Condition A	6.2 ± 0.6	6.9 ± 0.8	6.8 ± 0.9	< 0.001	0.59
Condition B	6.5 ± 0.8	6.4 ± 0.7	6.3 ± 0.8		
Condition C	6.3 ± 0.8	6.4 ± 0.6	6.1 ± 0.6		
cfPWV‐adjusted (m/s)					
Condition A	6.2 ± 2.3	6.9 ± 2.3	6.6 ± 2.3		
Condition B	6.5 ± 2.3	6.4 ± 2.3	6.3 ± 2.3	0.007	
Condition C	6.3 ± 2.3	6.5 ± 2.3	6.1 ± 2.3		
AIx (%)^a^ ^,^ ^c^					
Condition A	16.1 ± 5.3	30.6 ± 10.7	18.7 ± 7.8	< 0.001	0.42
Condition B	14.0 ± 6.2	20.5 ± 8.3	19.1 ± 11.3		
Condition C	14.5 ± 7.7	19.5 ± 6.8	16.8 ± 7.6		
AIx@75 (%)^b^					
Condition A	−6.4 ± 4.1	−1.0 ± 4.3	−1.2 ± 4.1	0.11	0.20
Condition B	−7.6 ± 3.2	−5.0 ± 2.9	−5.6 ± 2.3		
Condition C	−5.6 ± 5.1	−4.9 ± 8.6	−4.9 ± 4.1		
AP (mmHg)^a^					
Condition A	9.2 ± 4.0	23.5 ± 9.4	11.6 ± 6.3	< 0.001	0.49
Condition B	7.5 ± 4.1	13.4 ± 7.1	11.5 ± 8.0		
Condition C	7.6 ± 4.5	12.1 ± 5.5	9.2 ± 5.0		
cPP (mmHg)^a^					
Condition A	55.1 ± 8.8	75.6 ± 12.2	60.5 ± 10.8	0.02	0.23
Condition B	51.4 ± 5.2	61.5 ± 11.8	56.3 ± 8.9		
Condition C	50.3 ± 6.5	60.5 ± 9.6	53.1 ± 7.5		
cMAP (mmHg)^c^					
Condition A	82.5 ± 10.5	84.0 ± 10.2	80.3 ± 10.3	0.48	0.08
Condition B	84.8 ± 10.8	87.5 ± 13.1	81.6 ± 11.4		
Condition C	83.5 ± 12.5	88.5 ± 10.3	85.0 ± 10.9		
SEVR (%)					
Condition A	160.5 ± 22.3	142.9 ± 25.6	132.3 ± 28.6	0.14	0.18
Condition B	180.4 ± 51.2	155.6 ± 22.9	176.3 ± 55.5		
Condition C	162.0 ± 37.3	158.0 ± 35.0	161.3 ± 29.0		
pSBP (mmHg)^c^					
Condition A	119.0 ± 10.2	126.5 ± 12.9	119.6 ± 11.2	0.83	0.03
Condition B	120.0 ± 10.5	124.5 ± 13.4	119.7 ± 11.6		
Condition C	119.1 ± 10.4	124.5 ± 11.0	120.0 ± 11.1		
pDBP (mmHg)^a^					
Condition A	58.3 ± 9.3	47.9 ± 13.5	53.9 ± 10.4	0.03	0.22
Condition B	62.4 ± 9.2	58.7 ± 13.1	57.5 ± 9.7		
Condition C	62.3 ± 10.8	60.0 ± 9.9	61.7 ± 8.6		
HR (bpm)^b^ ^,^ ^c^					
Condition A	61.4 ± 8.5	72.4 ± 8.9	72.1 ± 8.4	0.12	0.19
Condition B	58.9 ± 6.7	64.2 ± 6.0	62.9 ± 4.9		
Condition C	63.1 ± 10.7	64.8 ± 17.7	64.4 ± 8.5		

*Note:* Data are displayed as means ± SD.

Abbreviations: AIx, augmentation index; Aix@75, augmentation index normalized at 75bmp; AP, augmentation pressure; cDBP, central diastolic blood pressure; cfPWV, carotid‐femoral pulse wave velocity; cfPWV‐adj., cfPWV adjusted for mean arterial pressure; cMAP, central mean arterial pressure; cPP, central pulse pressure; cSBP, central systolic blood pressure; HR, heart rate; pDBP, peripheral diastolic blood pressure; pSBP, peripheral systolic blood pressure; SEVR, subendocardial viability ratio.

^a^ Interaction main effect (*p* < 0.05).

^b^ Condition main effect (*p* < 0.05).

^c^ Time main effect (*p* < 0.05).

**TABLE 3:** Oxygenation variables.

	Time point		Condition effect	Interaction effect	
	Baseline	Recovery	*p* For condition effect	*p* For interaction effect	Effect size (η^2^_p_)
TSI (%)					
Condition A	67.3 ± 3.9	65.0 ± 7.0		0.43	0.08
Condition B	69.0 ± 2.4	67.4 ± 5.0			
Condition C	66.9 ± 5.7	68.5 ± 7.6			
TSI slope (%/s)					
Condition A		0.11 ± 0.22	0.14		0.17
Condition B		0.04 ± 0.39			
Condition C		0.25 ± 0.35			
tHb slope (AU/s)					
Condition A		0.06 ± 0.21	0.39		0.08
Condition B		−0.07 ± 0.18			
Condition C		0.06 ± 0.17			

*Note:* Data are displayed as means ± SD.

Abbreviations: AU, arbitrary units; tHb, total hemoglobin; TSI, tissue oxygen saturation.

Condition main effects were observed for AIx@75 $(\eta_{p}^{2}=0.27)$ and HR, $\left(\eta_{p}^{2}=0.26\right)$, (all *p* < 0.05) with higher values reported for Condition A than Condition B and Condition C.

Time main effects were evidenced for AIx@75 $\left(\eta_{p}^{2}=0.29\right)$, HR $(\eta_{p}^{2}=0.28)$, cSBP $\left(\eta_{p}^{2}=0.67\right)$, cMAP $(\eta_{p}^{2}=0.42)$, and pSBP $(\eta_{p}^{2}=0.51)$, where all variables significantly increased during recovery (all *p* < 0.05). There were no significant condition by time interactions or main effects for SEVR, TSI%, tHb, and TSI% upslope (all *p* > 0.05).

## 4 Discussion

This study examined acute vascular responses to three RT protocols with matched proximity to failure (2RIR) and rest intervals but varying in load and set or repetition volume. Specifically, we compared, low‐volume/moderate‐load/high‐repetition (Condition A), low‐volume/high‐load/low‐repetition (Condition B), and moderate‐volume/high‐load/low‐repetition (Condition C) protocols. Contrary to our first hypothesis, the Condition A protocol elicited significantly greater increases in cfPWV (from 6.2 ± 0.6 to 6.9 ± 0.8 m/s), AIx (from 16.1 ± 5.3 to 30.6 ± 10.7%), and AIx@75 (from −6.4 ± 4.1 to −1.0 ± 4.3%) compared to the heavy load‐low rep protocols (Condition B and Condition C), regardless of set or repetition volume, whereas no significant between‐condition differences were observed for muscle oxygenation variables.

The observed hemodynamic changes occurred despite set and repetition volume being matched and all sets being performed at a controlled proximity to failure, quantified using the RPE/RIR scale. In addition, the Condition C protocol, despite its higher set volume, did not significantly differ from Condition B protocol in cfPWV or other AS indices and did not increase cfPWV relative to baseline. Results presented herein indicate that even when effort is submaximal and consistent across conditions, lower load‐higher repetition RT protocols may elicit greater acute vascular responses than higher load‐lower repetition RT schemes, even when performed with low volume (i.e., 2 sets) and under conditions of equal set (i.e., Condition A vs. B) and total repetition volume (Condition A vs. C). Furthermore, the lack of significant differences in cfPWV and pulsatile hemodynamics between the Condition C and Condition B suggests a nonlinear relationship between overall volume and large artery stiffness. Lastly, the absence of significant differences in oxygenation variables implies that acute changes in localized tissue perfusion during RT may not directly reflect central macrovascular responses in the immediate postexercise period.

These findings support and expand upon our previous findings (Karanasios, Hannah, et al. 2025a) indicating that moderate‐load resistance training to volitional failure (12RM) elicits greater cfPWV and AIx responses than heavy‐load training (4RM) when proximity to failure is held constant. The present study demonstrates that even under submaximal effort conditions (2RIR), higher repetition numbers acutely increase arterial stiffness, indicating that RT variables, such as volume of repetition per set and time under tension (TUT), have a more pronounced effect on central hemodynamics rather than the load itself.

TUT is derived from the sum of durations of each phase of the lift (i.e., concentric, eccentric, and isometric) (Wilk et al. 2021). Herein, TUT for the Condition A and Condition C was approximately 40 and 16s, respectively. Due to the inverse relationship between load and repetitions (Willardson et al. 2010), lighter loads allow more repetitions to be performed under both maximal and submaximal effort. Maintaining repetition duration constant, higher repetitions schemes would result in a greater TUT and a prolonged set duration. Prolonged TUT increases metabolite accumulation and motor unit recruitment, elevating sympathoexcitation through activation of the exercise pressor reflex (Rúa‐Alonso et al. 2020). This reflex mechanism increases heart rate and cardiac contractility while also induces vasoconstriction in nonactive vascular beds, thus increasing total peripheral resistance (MacDonald 2002). The physiological responses elicited by these autonomic adjustments include the increase of left ventricular contractility and wave reflection originating from peripheral sites (Wakeham et al. 2025b).

The augmented forward wave pressure, driven by increased cardiac contractility, propagates through the aortic trunk, which when combined with reflected waves, results in an elevation of the late systolic peak, thereby directly increasing AP and AIx (Shirwany and Zou 2010). Concurrently, acceleration of the forward pressure wave and increased wave reflection from peripheral sites, results in a premature return of the reflected wave that elevates late systolic peak pressure, thus increasing cPP and elevating AP further (Bonarjee 2018; Wakeham et al. 2025b). Findings presented herein are in line with these observations. The Condition A condition led to a significant greater increase in HR, AP, cPP, and AIx immediately postexercise (post). Overall, these pulsatile hemodynamic responses possibly increased arterial wall distending pressure, leading to recruitment of stiff collagen fibers thereby elevating cfPWV (Wakeham et al. 2025b).

In support of this, recent evidence using heart rate variability indices suggests that longer TUT per set may delay parasympathetic reactivation during recovery compared to sets with shorter TUT and heavier load when volume, rest, and repetition duration remains constant, although direct measures of sympathetic nerve activity were not obtained in these studies (Güngör et al. 2025; Rúa‐Alonso et al. 2020). Similarly, previous reports indicate higher metabolite accumulation during sets with a higher number of repetitions even when training volume is equalized (Gorostiaga et al. 2014; Rúa‐Alonso et al. 2020). Heightened sympathetic activity and increased metabolite accumulation sustain vasoconstriction and delay postexercise hemodynamic recovery, potentially contributing to acute increases in AS (Holwerda et al. 2019). In addition, previous research performing continuous blood pressure monitoring during RT has reported a greater pressure response following 15RM versus. 4RM RT, demonstrating that blood pressure rises progressively with the number of repetitions, irrespective of the external load (Gjovaag et al. 2016). Considering the strong associations between blood pressure and cfPWV (Meani et al. 2018), it can be argued that a similar trend might be evident in acute cfPWV responses during RT. Yet, this is still speculative since current cfPWV evaluation is discontinuous by nature, limited to the magnitude of change between pre and postexercise.

Furthermore, the extended TUT in the Condition A protocol likely resulted in a more prolonged mechanical compression of the vasculature. During RT contracting skeletal muscles generate high intramuscular pressures, especially as fatigue accumulates (Wakeham et al. 2025a). Mechanical compression during RT may serve as a ‘functional’ site for wave reflection, similar to external compression cuffs. Research has shown that external compression applied to the calf increases the magnitude of pressure from wave reflections in the upstream femoral artery (Heffernan et al. 2013). These reflected waves elevate the late systolic peak pressure, increasing cPP and AP, and may induce transient arterial stiffening, reflected in elevated PWV.

In the present study pulse wave reflection measures were significantly higher immediately post exercise in Condition A than Condition B and Condition C. Increases in Aix from resting values were 81.2% in the Condition A at Post, versus. 46% and 34.35% in Condition B and Condition C, respectively. Values of AIx@75 as well were overall significantly higher in Condition A throughout the recovery period (condition effect), indicating that these effects were not solely heart rate dependent. Wave reflection indices are moderated by the velocity of the pulse wave (Davies and Struthers 2003), thus the elevated cfPWV in Condition A may have led to an accelerated return of the reflected wave, thereby increasing AIx. This observation is in accordance with Karanasios, Hannah, et al. (2025a) where AIx and AIx@75 increased significantly in the moderate load/high rep condition, suggesting a more pronounced wave reflection response with lower load/higher rep RT protocols.

Data presented herein showed that cSBP significantly increased immediately post, regardless the RT protocol. Increases from resting values were 9%, 5.7%, and 7.1% for Condition A, Condition B, and Condition C, respectively. Changes in cSBP may occur independent of changes in cfPWV (Figueroa et al. 2019), with conflicting results being reported following acute RT (Parks et al. 2020; Thiebaud et al. 2016). In addition, cPP was significantly higher in Condition A compared to Condition B and Condition C, with residual effects persisting up to 15 min into recovery. Central pulse pressure serves as an indicative index for arterial stiffness as it reflects the pulsatile component of blood pressure, influenced by both the elastic properties of large arteries and the reflected waves (Marti et al. 2012). This finding aligns with the higher AIx noted in the Condition A. Elevation of AIx indicates that the return of the reflected wave to the aorta occurs mostly during systole rather than early diastole, thereby increasing cSBP, reducing cDBP (Bonarjee 2018), and as a result elevating cPP. Since myocardial perfusion occurs during diastole, the premature return of the reflected wave could indicate a decrease in coronary blood flow (Bonarjee 2018). In the present study, cDBP was significantly lower in the Condition A compared to both Condition B and Condition C and significantly decreased during recovery. This finding is in line with some (Karanasios, Hannah, et al. 2025a; Thiebaud et al. 2016) but in contrast to others (Parks et al. 2020). Notably, changes in SEVR, reflecting myocardial perfusion, were not statistically different across conditions, although the reduction was more pronounced in Condition A (17.5%) compared with Condition B (2.2%) and Condition C (0.4%). Our prior study indicated significantly lower SEVR in moderate load (12RM) versus heavy load (4RM) protocols, when both RT protocols performed to volitional failure. This suggests that proximity to failure may be a stronger determinant of coronary perfusion as measured by SEVR. Yet, due to the scarcity of available data, future research is needed to validate this notion.

Contrary to our second hypothesis, Condition C, despite being higher in set and overall volume load, did not promote greater increases in cfPWV or AIx compared to Condition B. This suggests that overall volume may not independently increase acute arterial stiffness when the number of repetitions per set is kept low (e.g., ≤ 4). Notably, a previous study reported no cumulative effect in blood pressure following high load/low repetition RT (4RM) from the first to third set. Conversely, during low load/high repetition (15RM) RT exercise, peak blood pressure in set 3 was significantly elevated compared to set 2 (Gjovaag et al. 2016). Given the paucity of available data on AS indices, future research is needed to further explore the influence of RT volume on acute macrovascular responses.

Findings presented herein indicate no differences in microvascular responses between the RT protocols adopted, suggesting that acute increases in AS can occur independently of changes in microvascular tissue perfusion. Previous studies have reported impaired microvascular reactivity as indexed be slower reperfusion rate (i.e., smaller TSI upslope) within cohorts of different vascular profiles, such as hypertensive (Dipla et al. 2017), obese (Soares and Murias 2018) and older adults at risk of CVD (Oliveira et al. 2021). Nonetheless, the young healthy participants of the current study likely possessed preserved microvascular function and oxidative capacity thus limiting any detectable changes. In addition, those studies largely employed vascular occlusion tests, which generate a strong and uniform hyperemic stimulus, primarily driven by downstream metabolic vasodilation after cuff release (McLay et al. 2016). In contrast, postexercise hyperemia during dynamic RT is generated from muscle contraction, yielding alternating phases of blood flow restriction and dilation (e.g., compensatory circulation during the rest periods). Mechanical compression of intramuscular blood vessels from repeated contractions can transiently restrict flow, leading to a recovery pattern that is influenced by the magnitude of these compressions rather than by a single uniform ischemic release (Alvares et al. 2020). Hence, oxygenation kinetics after acute RT may follow distinct recovery patterns than previous studies utilizing vascular occlusion tests.

Collectively, the current findings demonstrate that even at a controlled submaximal effort level, the repetition scheme itself affects acute vascular responses, independent of the load lifted or total set volume. These findings contrast with previous assumptions that high‐load RT increases AS (Miyachi et al. 2004; Zhang et al. 2021). Current findings highlight the importance of other RT variables such as proximity to failure and TUT. From a practical perspective, repetition configuration appears to influence acute vascular stress during RT. Although the reported increases in AS were observed in healthy adults, practitioners working with individuals at elevated cardiovascular risk may consider favoring lower‐repetition schemes to reduce acute pulsatile load.

Some limitations of the current study must be acknowledged. The RPE/RIR‐based method used to quantify and equate proximity to failure is inherently subjective and prone to over/under estimation (REF). Nonetheless, our data revealed highly accurate estimations across sessions (Average RPE 8.0 ± 0.3 and 7.9 ± 0.3, for the hex deadlift and bench pull, respectively), strengthening the validity of our standardization. In addition, findings observed herein are specific to the RT protocols implemented (i.e., the number of sets and exercises and rest duration). Hence, it remains uncertain whether similar vascular and oxygenation responses would be observed under different RT schemes. Lastly, the acute nature of the intervention limits extrapolation to chronic training adaptations. Future research should examine the long‐term effects of various resistance training configurations on vascular health and investigate whether the acute vascular responses identified herein are predictive of chronic arterial adaptations. Despite these limitations, this study offers valuable insights regarding the effects of RT variables, such as load and volume, on macro and microvascular responses.

## 5 Conclusion

This study demonstrates that moderate load/high repetition RT exercise induced a more pronounced hemodynamic response compared to heavy‐load low‐repetition RT protocols, independent of total set volume. The observed vascular response occurred without parallel changes in local muscle oxygenation. Moreover, increasing RT volume through a higher number of sets did not increase arterial stiffness when the number of repetitions per set are kept low (< 5). These results emphasize the importance of the number of repetitions per set and TUT in mediating acute vascular responses to RT.

## Funding

The authors have nothing to report.

## Ethics Statement

The study was approved by the Faculty of Health and Wellbeing of the University of Winchester (HWB_REC_240_Karanasios). Experimental procedures were conducted following the approved ethics submission document. All participants received written information explaining the procedures and purpose of the study and gave their written consent prior to data collection.

## Conflicts of Interest

The authors declare no conflicts of interest.

## Data Availability

The data that support the findings of this study are available from the corresponding author upon reasonable request.
